# Simulating the Distribution of Individual Livestock Farms and Their Populations in the United States: An Example Using Domestic Swine (*Sus scrofa domesticus*) Farms

**DOI:** 10.1371/journal.pone.0140338

**Published:** 2015-11-16

**Authors:** Christopher L. Burdett, Brian R. Kraus, Sarah J. Garza, Ryan S. Miller, Kathe E. Bjork

**Affiliations:** 1 Colorado State University, Department of Biology, Fort Collins, Colorado, United States of America; 2 United States Department of Agriculture, Animal and Plant Health Inspection Service, Veterinary Services, Centers for Animal Health and Epidemiology, Fort Collins, Colorado, United States of America; National Institute for Public Health and the Environment, NETHERLANDS

## Abstract

Livestock distribution in the United States (U.S.) can only be mapped at a county-level or worse resolution. We developed a spatial microsimulation model called the Farm Location and Agricultural Production Simulator (FLAPS) that simulated the distribution and populations of individual livestock farms throughout the conterminous U.S. Using domestic pigs (*Sus scrofa domesticus*) as an example species, we customized iterative proportional-fitting algorithms for the hierarchical structure of the U.S. Census of Agriculture and imputed unpublished state- or county-level livestock population totals that were redacted to ensure confidentiality. We used a weighted sampling design to collect data on the presence and absence of farms and used them to develop a national-scale distribution model that predicted the distribution of individual farms at a 100 m resolution. We implemented microsimulation algorithms that simulated the populations and locations of individual farms using output from our imputed Census of Agriculture dataset and distribution model. Approximately 19% of county-level pig population totals were unpublished in the 2012 Census of Agriculture and needed to be imputed. Using aerial photography, we confirmed the presence or absence of livestock farms at 10,238 locations and found livestock farms were correlated with open areas, cropland, and roads, and also areas with cooler temperatures and gentler topography. The distribution of swine farms was highly variable, but cross-validation of our distribution model produced an area under the receiver-operating characteristics curve value of 0.78, which indicated good predictive performance. Verification analyses showed FLAPS accurately imputed and simulated Census of Agriculture data based on absolute percent difference values of < 0.01% at the state-to-national scale, 3.26% for the county-to-state scale, and 0.03% for the individual farm-to-county scale. Our output data have many applications for risk management of agricultural systems including epidemiological studies, food safety, biosecurity issues, emergency-response planning, and conflicts between livestock and other natural resources.

## Introduction

Models are essential to our understanding of complex economic and ecological systems [1]. However, the scales at which models can be developed, and the subsequent problems they can inform, are often limited by our inability to model interactions at the finest spatial, temporal, or organizational resolutions. Advances in remote-sensing technology now allow environmental characteristics like climate, land cover, and topography to be measured at fine spatial resolutions. However, fine-grained data measuring human socioeconomic activities are often unavailable, often because they contain confidential or proprietary information.

Data describing livestock and poultry (hereafter livestock) production in the United States (U.S.) are an example. The most comprehensive data on livestock production in the U.S. are from the Census of Agriculture, which is conducted every five years by the U.S. Department of Agriculture, National Agricultural Statistics Service (NASS). While farms and ranches earning > $1,000 in a census year are required to participate, U.S. law (Title 7 Chapter 55 Section 2276, U.S. Code of Federal Regulations) ensures the confidentiality of their responses. Consequently, unlike other countries, national-scale data depicting the locations and population sizes of individual livestock farms are not available for the U.S. The Census of Agriculture instead publishes aggregate data for counties and states. However, NASS must still redact aggregate data from counties or states with few farms, or when comparisons with other counties or states would reveal confidential information. The legal requirement for confidentiality means aggregate Census of Agriculture data for livestock are more complete and accurate in regions with large industries.

The lack of a national dataset of individual farm locations and populations negatively impacts the ability of agencies to manage serious animal and human health risks in the U.S. The characteristics of individual farms, especially their distances to other farms and the size of the populations on those farms, are needed to parameterize spatial epidemiological models [2]. While knowledge from previous outbreaks can inform the parameterization of these models and overcome coarse information about the spatial distribution of farms [3], such knowledge is not always available or accessible to risk managers. Further, the ability to plan effective response plans for other risks like natural disasters would benefit from access to data for individual farms.

A solution to this problem is to develop a model that simulates production on farms in the U.S. Microsimulation models are an ideal methodological choice since they have been used to disaggregate data in other fields like urban and transportation planning, social science, and business marketing [4–6]. However, there are several challenges to developing a microsimulation model using U.S. Census of Agriculture data.

The initial challenge is how to impute the redacted data. While there are many techniques for imputing missing data [7], they can be difficult to apply without sacrificing some of the inherent information embedded in census data. Most notably, functional mathematical relationships exist between hierarchical levels of census data where county totals must sum to state totals, and state totals must sum to national totals. Data that may be unpublished at a fine resolution (e.g., county) may be included in data published at coarser resolutions (e.g., state or national). Also, the Census of Agriculture publishes data describing both livestock population size and the number of farms. While the livestock population of a county or state can be redacted to maintain confidentiality, the number of farms is not confidential information and is always published. Knowing the corresponding number of farms at all hierarchical levels of the Census of Agriculture means any unpublished population data have a finite range of possible values. An effective imputation technique for Census of Agriculture data must be flexible enough to reconcile unpublished data with published data at higher hierarchical levels and incorporate farm counts into its imputation algorithms.

Another challenge is how to predict the locations of individual farms. While production in many agricultural industries clusters due to economies of scale [8,9], these broad-scale spatial patterns are already captured in aggregate census data. In contrast, the factors influencing the distribution of farms at finer spatial grains (e.g., < 1 km) are poorly understood. For example, are farms typically located closer to roads, or are farms randomly distributed relative to roads? Some livestock simulation models have predicted farm locations with subjective rules for where farms may be placed on a landscape [10–13]. It is unknown how well such a rule-based approach might perform relative to a probabilistic approach that includes geographic information about the characteristics of individual farms.

A third challenge is how to disaggregate, or downscale, Census of Agriculture data to simulate both the locations and production levels of individual farms. Simulating livestock populations on individual farms is a relatively simple problem once all aggregate county-level population data have been imputed. However, this simulation model also must be spatially-explicit, or capable of simulating the locations of individual farms on real U.S. landscapes. This simulation model must therefore be linked to information about the geography of individual farms.

Several previous studies have simulated the spatial distribution of livestock. The Food and Agriculture Organization of the United Nations developed a model that depicts the distribution and abundance of livestock at a global scale [14]. Other simulated livestock population models have been developed at finer national or sub-national scales [10–12,15]. However, these studies either do not include the U.S., are too coarse grained to depict the locations of individual farms, or do not include information about the size of livestock populations on individual farms. Recently, a rule-based model was developed that simulated the distribution of poultry farm locations in the U.S. using Census of Agriculture data [13]. However, we are unaware of any previous studies that combined large empirical datasets of farm locations with probabilistic distribution models to simulate the locations and populations of individual livestock farms in the U.S.

The goal of this study was to develop a livestock-distribution and population-simulation model that could depict the locations and production levels of individual livestock farms throughout the conterminous U.S. We named our model the Farm Location and Agricultural Production Simulator (FLAPS). The FLAPS model utilizes data from the Census of Agriculture, in conjunction with probabilistic and microsimulation models, to simulate the distribution of farms and their livestock populations at a high spatial resolution (100 m) throughout the conterminous U.S. We demonstrate our modeling methodology using domestic pigs (*Sus scrofa domesticus*) as an example livestock species.

## Methods

### The FLAPS model

**Fig 1:**
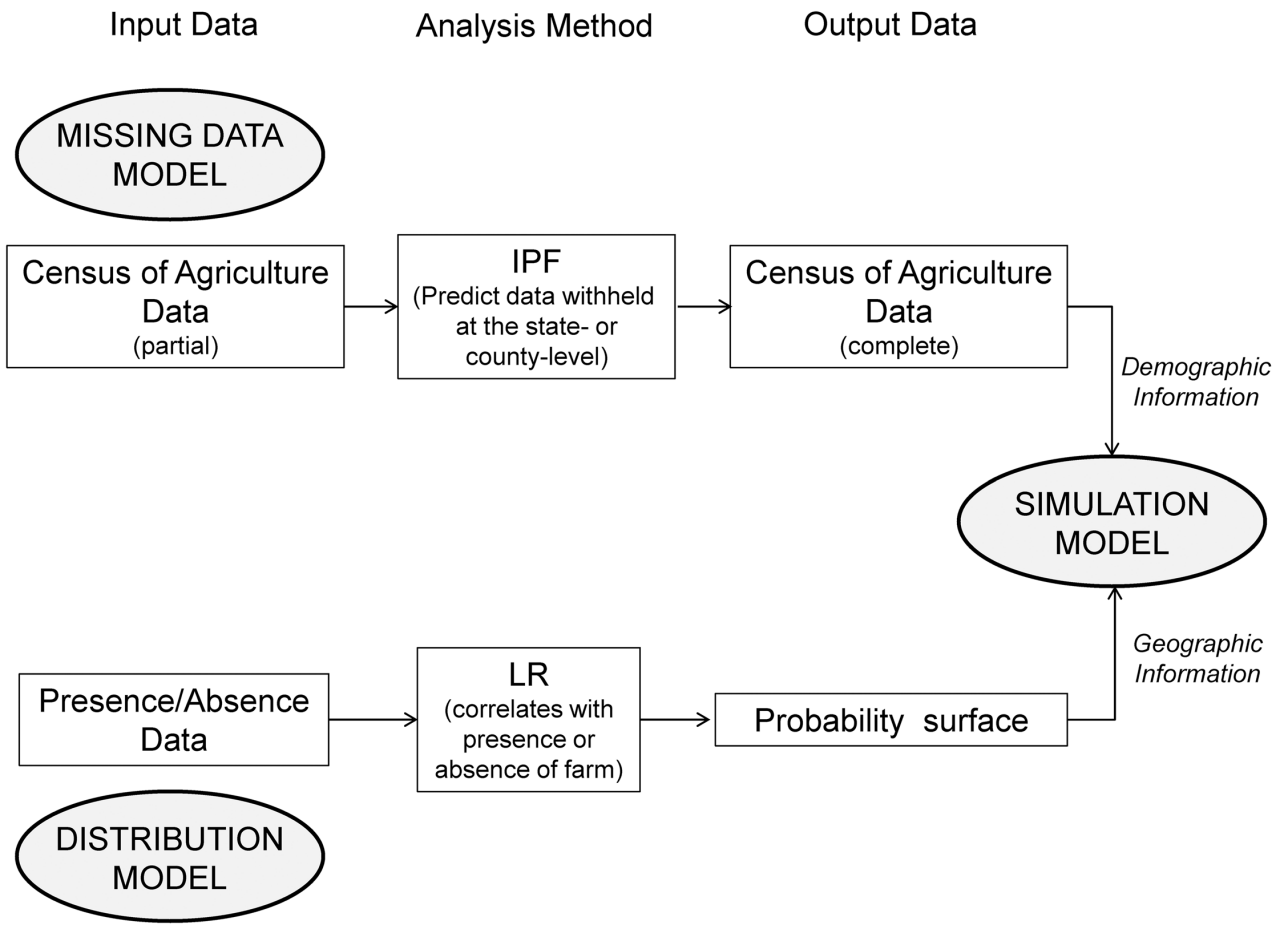
The structure of the FLAPS model. The FLAPS population simulation model consists of three interactive sub-models: (1) a missing-data model, (2) a distribution model, and (3) a simulation model. The output of the missing-data and distribution models provides input data for the simulation model. The definitions of the acronyms are: (1) IPF = iterative proportional fitting, and (2) LR = logistic regression.

The FLAPS model contains three interactive sub-models that address the previously identified challenges of imputing missing data, predicting the geographic distribution of individual farms, and simulating populations on those farms. A useful way to interpret these sub-models is to recognize that the distribution and missing-data models input geographic and demographic information into the simulation model, which disaggregates those input data to create a synthetic population of individual farms (Fig 1).

The FLAPS model is spatially-explicit. The maximum extent of its output data is the conterminous U.S., and its minimum extent is an individual county. Regardless of whether FLAPS is run at a national, state, or county scale, output data are records with fields simulating the livestock population and latitude and longitude of individual farms at a 100 m spatial resolution.

### Census of Agriculture Data

**Fig 2:**
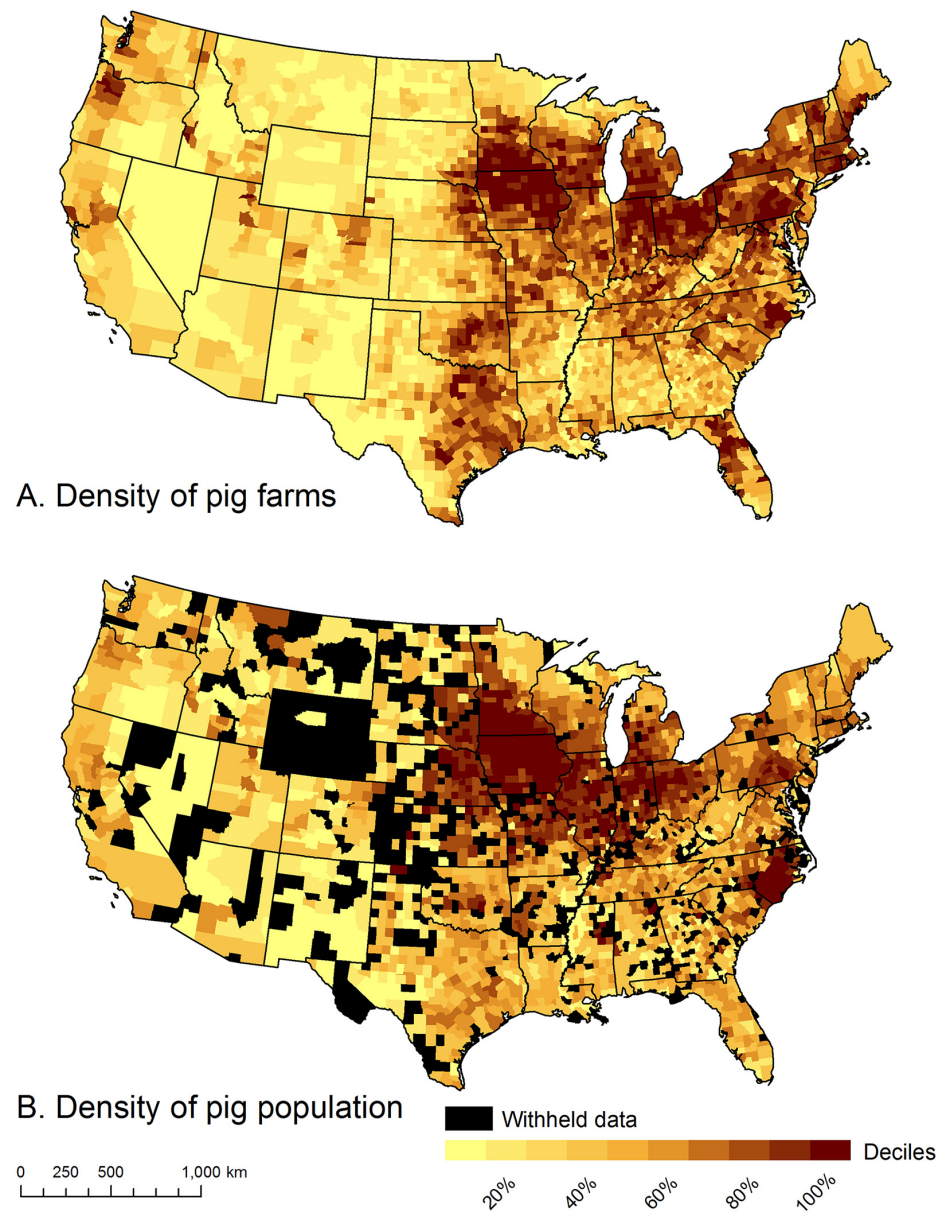
The density of domestic swine (A) farms, and (B) populations in the conterminous United States. Data are from 2012 Census of Agriculture [16]. Counties colored black in (B) are those counties where swine population data were withheld to ensure respondent confidentiality.

We collected publically available data from the 2012 U.S. Census of Agriculture [16]. This data contained the number of swine farms and the number of individual pigs for five hierarchical administrative units (national totals, state totals, state subtotals, county totals, and county subtotals). States and counties were uniquely identified by their Federal Information Processing Standard (FIPS) code numbers. Again, data describing the number of farms are not confidential information, and were published for all administrative units (Fig 2A); only data describing the number of individual pigs (i.e., population size) were unpublished when such information could be used to determine the number of animals on any individual farm (Fig 2B).

The state and county subtotals were the key information that allowed us to disaggregate Census of Agriculture data to individual farms. Subtotals described the number of farms and the pig-population sizes as frequency distributions with seven population-size categories: (1) 1–24, (2) 25–49, (3) 50–99, (4) 100–199, (5) 200–499, (6) 500–999, and (7) ≥ 1000 pigs. In addition to the 19% of U.S. counties whose county totals were unpublished (Fig 2B), 66% of U.S. counties had unpublished data in at least one bin of their population frequency distribution. The typical patterns of these state- or county-level frequency distributions were similar to those occurring nationally (Table 1) where the majority of swine farms have small populations, but most of the population occurs on a small number of large farms.

**Table 1:** Example of Census of Agriculture data from 2012 for the entire U.S. (including Alaska and Hawaii) showing the paired nature of the frequency distributions for the number of swine farms and individual pigs. The number of swine farms is not confidential information and is published for all hierarchical levels of the Census of Agriculture. In contrast, the number of individual pigs can reveal socioeconomic information about individual farms and can be redacted, most commonly for county totals and subtotals due to fewer farms in these finer resolution categories.

				Farm/population-size class^a^				
Data type	*n* ^b^	1 to 24	25 to 49	50 to 99	100 to 199	200 to 499	500 to 999	≥ 1000
Farm	63,246	41,688	3,435	2,161	1,469	2,115	1,977	10,401
Population	66,026,785	244,250	116,808	146,967	201,460	683,977	1,384,921	63,248,402

^a^ The total number of farms or population occurring within each of seven farm/population-size bins. Data from Table 19, 2012 U.S. Census of Agriculture [16].

^b^ Grand totals for the farm and population data types representing the total number of swine farms and total swine population for the entire U.S.

### Missing-data model for predicting unpublished Census of Agriculture data

We predicted missing data in the U.S. Census of Agriculture with iterative-proportional-fitting (IPF) algorithms, which are widely used in microsimulation models [4,6]. Iterative-proportional-fitting algorithms adjust an *n*-dimensional matrix until the marginal row (*Q*_*i*_) and column (*Q*_*j*_) totals equal pre-defined values. The pre-defined values used in IPF traditionally come from another dataset, often disaggregated microdata from a small sample of individuals or a survey [6]. Microdata are required for most applications of IPF in microsimulation models because researchers want to estimate values across multiple cross-classified variables. For example, researchers using human census data may wish to microsimulate data across multiple variables like age, sex, and income [6]. In contrast, we only needed to impute the single variable of livestock population. This simplification, coupled with knowing the number of farms and the size of the unknown population in each state or country, allowed us to estimate realistic seed values for unpublished data in the margins or body of our IPF matrices without microdata.

The hierarchical structure of the Census of Agriculture required us to implement IPF algorithms in two steps, first estimating missing state-level totals and subtotals and then applying the resulting complete but simulated state-level frequency distributions as inputs to estimate missing county-level data. For brevity, we demonstrate our IPF calculations for counties.

We used two types of pre-defined values in our data matrix. If data were available in the Census of Agriculture, we used the published county-level swine population totals for our predefined row totals (*Q*_*i*_), and the published state-level population totals for our pre-defined column totals (*Q*_*j*_). If the data were unpublished in the Census of Agriculture, we imputed seed values for any missing state totals, county totals, or county subtotals (i.e., the individual bins of the county-level swine population frequency distribution). We seeded realistic values (${\tilde{p}}_{ij}$) to speed convergence of the IPF algorithms. We defined seed values from the midpoints of the frequency-distribution bins, which we calculated by multiplying the number of farms in each bin by the maximum and minimum bin sizes. We defined the maximum bin size for the largest unbounded bin as the maximum population size of each county, which allowed for the simulation of the largest possible farms. These calculations defined absolute ranges for any unpublished population data in the frequency distribution bins. Defining a matrix element in row *i* and column *j* as *p*_*ij*_, the input values in our matrix would be:

*p*_*ij*_ = *p*_*ij*_, if the value was published in the Census of Agriculture

$p_{ij}={\tilde{p}}_{ij}$, if the value was imputed with a seed value

For a two-dimensional matrix where *p*_*ij(k)*_ is the matrix element in row *i*, column *j*, and iteration *k* and *Q*_*i*_ and *Q*_*j*_ are the pre-defined row and column sums [17], revised cell values are iteratively estimated with these equations:

$$p_{ij(k+1)}=\frac{p_{ij(k)}}{\sum_{j}p_{ij(k)}}\times Q_{i}$$ (1)

$$p_{ij(k+2)}=\frac{p_{ij(k+1)}}{\sum_{i}p_{ij(k+1)}}\times Q_{j}$$ (2)

Eqs (1) and (2) are employed iteratively to attempt to minimize the difference between the row sum and row-marginal total, and the column sum and column-marginal total. We allowed the algorithms to run for *m* iterations until the sum-marginal differences:

$$\sum_{j}p_{ij(m)}=Q_{i}\;\text{and}\;\sum_{i}p_{ij(m)}=Q_{j}$$ (3)

were zero, or until *m* = 2000. The IPF algorithm has been widely used in spatial microsimulation modeling because of its positive characteristics of convergence, preservation of correlation structures between attributes represented in the initial cell proportions, and ability to produce maximum-likelihood estimates of cell proportions [17,18]. However, the primary advantage of using IPF algorithms in this study was because they were flexible enough to incorporate the hierarchical structure of our census data.

### Distribution model for predicting geographic distribution of individual farms

We used a weighted random-sampling methodology to collect information about the features correlated with the fine-grained (e.g., ≤ 100 m) distribution of individual farms. We weighted our sampling design using county-level data from the 2012 Census of Agriculture. Specifically, we determined the proportion that each U.S. county contributed to the total number of swine farms in the U.S. and then multiplied that proportion by our desired sample size (*n* = 10,000) so that more sample points were collected from counties with more swine farms (Fig 2). Sample points were randomly distributed within the boundaries of their county.

Technicians inspected the sample points over aerial photographs in the ArcGIS 10.1 [19] geographic information system (GIS) to determine whether a livestock farm was present or absent at each sample point. We created a 1 km^2^ grid that covered the conterminous U.S. to restrict the distance over which we searched for farms near sample points. If no farm was found within the individual 1 km^2^ grid cell containing a sample point, the sample point was not moved and classified as an absence point. If a farm having livestock infrastructure (S1 Text) occurred within a grid cell, the point was moved in the GIS to the center of the barn, corral, or pasture where livestock were most likely to occur and classified as a presence point. Additional farms opportunistically found in adjacent grid cells were included in our sample to increase the number of presence points and improve our ability to discriminate between the factors correlated with farm presence and absence. The aerial images lag approximately 1–2 years behind the current date [20], so they generally coincided with the 2012 Census of Agriculture.

Although it was impossible to be certain of the livestock species present on any individual farm, we interviewed U.S. Department of Agriculture livestock experts and developed a decision tree to classify our sampling data into the most likely species produced at each farm (S1 Text). However, we did not use these species-level classifications in our analyses since their differences were generally below the 100 m resolution of the FLAPS model (S2 Text).

The predictor variables for our distribution model included environmental features like land-cover categories, topography, and climate, and anthropogenic features like roads and urban markets (Table 2). We noticed during sampling that presence points (i.e., farms) were frequently near roads or the edges of certain types of land cover, so we measured the anthropogenic and land-cover covariates as the distances from these features to our presence-absence response points. Topography and climate did not need to be interpreted as distances, so we represented the prevailing topographic and climate conditions at 90 m and 1 km resolutions surrounding the sample points. We evaluated quadratic forms of all covariates to detect non-linear relationships which might be particularly important for our distance-based covariates. We chose to use either the linear or quadratic form of each covariate based on which form had the lower AIC score in comparisons using simple logistic-regression models.

**Table 2:** Covariates used to model the distribution of swine farms in the United States.

Covariate^a^	Description
**Land-cover categories** ^**b**^	
*d*Barren	Barren land
*d*Crop	Cropland
*d*Develop	Developed areas (low, medium and medium-high intensities)
*d*Forest	Upland forest
*d*Grass	Grassland
*d*Open^c^	Open areas
*d*Pasture	Pasture
*d*Shrub	Shrubland or scrubland
*d*Urban	Developed areas (high intensity)
*d*Water	Water
*d*Wetland	Lowland areas
**Topography** ^**b**^	
Slope	Slope (measured in degrees)
Rugged	Ruggedness (measurement of topographic variation)
**Climate** ^**b**^	
Temp	Mean annual temperature (1950–2000)
Precip	Mean annual precipitation (1950–2000)
**Transportation** ^**b**^	
*d*Roads	Roads

^a^ Covariates with prefix *d* are measured as linear distances (m) to the environmental or anthropogenic feature.

^b^ Sources and references: Land-cover categories: 2006 National Land Cover Dataset [21]; Topography: National Elevation Dataset [22]; Climate: WORLDCLIM database [23]; Transportation: Environmental Systems Research Institute (ESRI) World Transportation [24].

^c^ Open areas = Cropland + Pasture + Grassland + low and medium intensity Developed areas

We used an information-theoretic inferential framework to develop our distribution model. To avoid multicollinearity, we examined a Spearman correlation matrix of all our covariates and did not use correlated variables (|*r*| ≥ 0.7) in the same candidate model. When correlations were found, we chose the covariate that was more widely available as spatial data or more likely to be associated with the presence of farms. We created a global model using all uncorrelated variables, fit all subsets of this global model, and ranked models by their AIC_*i*_Δs relative to the model with the lowest AIC value. Although an all-subsets approach can be considered data dredging when prior knowledge allows a more thoughtful set of candidate models, the approach can be useful when the research problem is poorly understood [25], as is the case with the spatial distribution of individual farms. To minimize any model-selection bias resulting from our all-subsets approach, we used multi-model inference across all models with an AIC_*i*_Δ ≤ 2 to estimate model-averaged regression coefficients ($\tilde{\bar{\beta }}$) for our final distribution model.

We mapped the logistic-regression equation for this final model in ArcGIS 10.1 to create a probability surface depicting the probability of occurrence for swine farms in the conterminous U.S. We then refined this surface by incorporating a mask layer that prohibited farms from being placed in urban areas and water bodies [21] or public lands [26].

### Simulation model for disaggregating swine populations to individual farms

Our missing-data model output a complete but simulated frequency distribution depicting swine production for each U.S. state and county. Our distribution model then provided a spatial probability surface to estimate the locations of individual farms. The final component of the FLAPS model was a simulation model that used the output from these two models to disaggregate the county-level demographic data and simulate both the locations and populations of individual farms.

**Fig 3:**
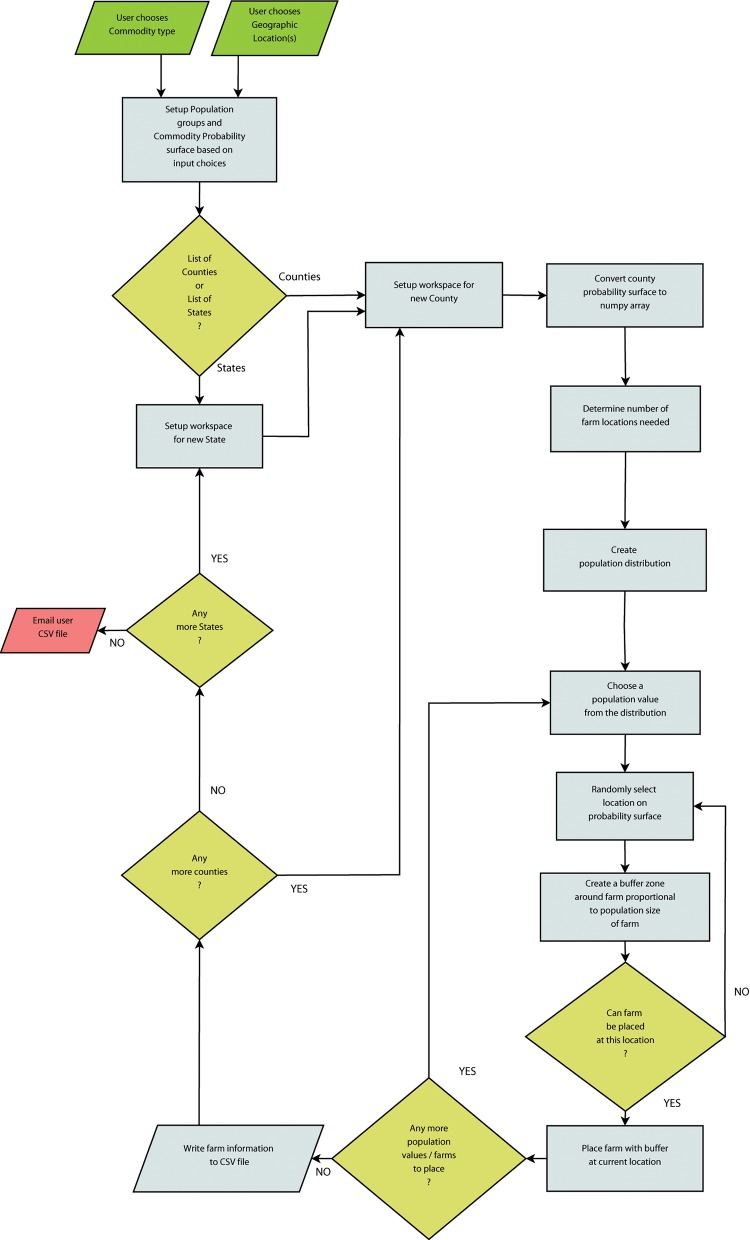
A flow chart of iterative steps in the simulation model based on algorithms used to place individual farms with both geographic (i.e., location) and demographic (i.e., population) attributes.

We designed the simulation model as a tool with a graphical-user interface so end users could request data for their specific area of interest. The algorithms used to develop this user interface can be summarized in a flow chart (Fig 3). The key function of the simulation model was to create the disaggregate population distribution. This process involved three steps: (1) creating an initial disaggregate dataset by downscaling aggregate populations values to the number of farms in each bin of the county-level frequency distributions, (2) correcting the initial disaggregate data with IPF algorithms so farms were bounded within the ranges of their population-size bin, and (3) attaching a geographic location to the disaggregated populations values representing individual farms.

The simulation model began by generating an initial disaggregate dataset of population values for individual farms. This was done by assuming the swine populations on farms in each of the seven bins of the population frequency distribution would follow a uniform distribution *U*(*a*,*b*) where *a* and *b* were the minimum and maximum bin values. We chose the uniform distribution to disaggregate our data since it is a null distribution that does not assume any data structure beyond the values for the minimum and maximum bounds [27], and we had no reason to expect the population values would have a consistent structure within bins. For the largest unbounded bin we set the maximum bin boundary to the total unknown population for that county.

The second step in creating disaggregate population values used IPF algorithms to ensure our disaggregated data summed to the aggregate output from our missing-data model. While the IPF algorithms for the missing-data model operated on a two-dimensional matrix, the IPF algorithms for the simulation model utilized only Eq (1) and operated on a one-dimensional row of data that were the initial disaggregate values created in step one. Values within the distribution were increased or decreased depending on whether the current row sum was less than, or greater than, the marginal row total. Values near the boundaries of the distribution sometimes were outside their known bin, which was defined by the frequency distributions for number of farms, by 1–2 animals due to rounding errors. We corrected these errors by readjusting them toward the bin midpoint. This adjustment process was iteratively fit until the differences between the sum of the disaggregate data and aggregate output from the missing-data model were minimized.

Finally, after populations were disaggregated to individual farms, we attached a geographic location (i.e., coordinates for latitude and longitude) to each farm using the probability surface from our distribution model. Our probability surface was a spatially-explicit raster dataset consisting of 100 m pixels where each pixel’s value represented the probability of livestock-farm occurrence. We defined a frequency distribution of occurrence probabilities on which to place farms using the predicted values for the presence locations from our model-averaged logistic-regression model. Using this entire range of probability values allowed us to better reflect that farms did not always occur in locations with high probabilities of occurrence. We also restricted farms from being placed too close together by setting neighborhood boundaries around larger farms. Although we did not create boundaries for the smallest farms in the 1–24 population-size category, we created neighborhood boundaries of 25 pixels for the largest farms in the ≥ 1000 population-size category, and nine pixels for intermediate-sized farms in the five intermediate population-size categories. This step of adding locations to the individual farm populations began with the largest, unbounded group of farms with populations of ≥ 1000, and concluded with the smallest group of farms with 1–24 pigs.

All code for missing-data and simulation models was written in Python v2.7 and Numpy v1.6. These platforms were chosen because of their ability to interface with the ArcGIS 10.1 software we used for geospatial calculations and our graphical-user interface [19]. Statistical analyses associated with the distribution model were conducted in the software packages R [28] and Stata [29].

### Model verification and evaluation

Validation of spatial microsimulation models is difficult because, like FLAPS, they are usually developed to depict data that don’t exist [30]. No independent national-scale livestock population data were available to validate FLAPS with external data. We therefore verified the proper functioning and evaluated the predictive performance of our model internally using the same datasets used for model-building. Specifically, we used verification analyses to demonstrate that the algorithms for our missing-data and simulation models were correctly implemented and operated as intended [31] and evaluated the predictive performance of our distribution model with a k-fold cross-validation that partitioned our model-building data into separate training and testing datasets [32].

We conducted two verification analyses for our missing-data model. First, we conducted two reaggregation analyses to measure how effectively the output from our IPF analyses reaggregated national totals from state totals and state totals from county totals. We conducted 100 iterations of both reaggregation analyses and measured performance with the absolute percentage difference metric, which has been previously used to evaluate synthetic population data [33]. Second, we conducted a substitution analysis of our IPF algorithms where we reclassified our simulated values (i.e., unpublished data) as published data then randomly selected the same number of values from the original published data, redacted their values, and reclassified them as unpublished data. The objective of this analysis was to demonstrate that our IPF algorithms would impute similar values throughout the data matrix regardless of the specific pattern of missing data. We randomly redacted data from our complete but simulated county-level Census of Agriculture dataset 100 times and used the mean absolute percent difference across these iterations to measure the performance of the substitution analysis.

We verified our simulation model with a reaggregation analysis too. The objective of this analysis was to demonstrate that our final output data of individual farm populations summed to the county totals from which they were disaggregated. We created 20 national-scale runs of FLAPS, reaggregated the individual-farm output to a county-level, and calculated the mean absolute percent difference between the reaggregated values and original county-level values for each of the 20 iterations. We averaged values for all counties and presented results at a state level.

Lastly, we evaluated our geographic model with a k-fold cross-validation procedure that split the input data into separate training and testing datasets. This procedure was repeated for five iterations (i.e., folds) using a random selections of 80% of the input data to train the model and the remaining 20% to test the model’s predictive performance. We evaluated predictive performance with area under the receiver-operating characteristic curves which we averaged across the five folds of the cross-validation procedure. We also calculated variable importance ranks to detect influential covariates.

## Results

### Missing-data model for predicting unpublished Census of Agriculture data

Our missing-data model provided us with a complete, but simulated, county-level Census of Agriculture dataset for swine farms and populations in the conterminous U.S. An example of the iterative steps of the IPF algorithm applied to a hypothetical Census of Agriculture table at the county level is provided (S1 Presentation).

**Fig 4:**
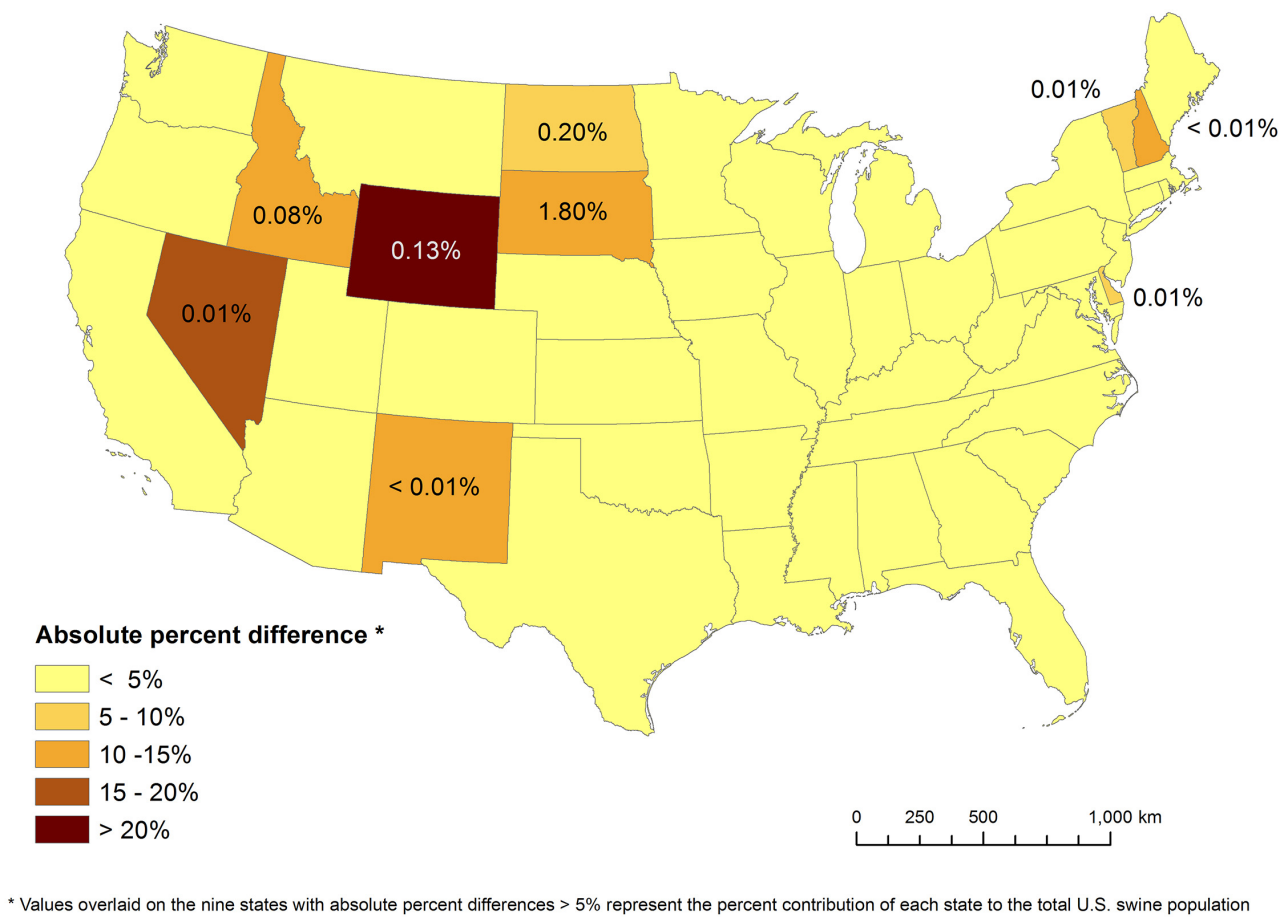
Mean absolute percent differences for states in our county-to-state IPF verification analysis. This map depicts the reaggregation of our county-level estimates of swine populations to the state-level totals from which they were derived. The most missing data in the Census of Agriculture occurs at the county-level, and this missing data precludes the high accuracy (mean absolute percent differences of ≤ 0.03%) our IPF algorithms achieved at the other two hierarchical scales (individual farms to counties, and states to the national total). For the nine states with absolute percent differences of > 5%, we overlaid the percent of total U.S. swine population occurring in each state. Collectively, these nine states comprised only 2.24% of the total U.S. swine industry.

Our verification analyses provided excellent results at all scales. The state- to national-level reaggregation analysis indicated our IPF algorithms were nearly perfect; a rounding error allocated one extra pig among the 66,026,788 million pigs present in the U.S. in 2012. The county- to state-level reaggregation analysis was less efficient due to greater amounts of unpublished data but still had an absolute percent difference of only 3.14 ± 1.14% (mean ± SE) when averaged across the U.S. (Fig 4). States with larger industries had smaller absolute percent differences because they contained less unpublished data. The five states with the most sales in 2012 (Iowa, North Carolina, Minnesota, Illinois and Indiana) had a mean absolute percent difference of 0.03 ± 0.01% (mean ± SE). In contrast, nine states had absolute percent differences > 5.00% (Delaware, Idaho, Nevada, New Hampshire, New Mexico, North Dakota, South Dakota, Vermont, and Wyoming) (Fig 4). However, these nine states comprised only 2.24% of the U.S. swine industry, with South Dakota accounting for 1.80% of that amount.

Our IPF substitution analysis indicated the IPF algorithms for our missing-data model imputed similar final values across 100 different patterns of missing Census of Agriculture data. The mean absolute percent difference across all states was < 0.01%, and no state had an absolute percent difference > 0.20%.

### Distribution model for predicting geographic distribution of individual farms

Our sample contained the 10,000 records from our weighted random sampling and an additional 238 presence points that were opportunistically obtained during sampling. The overall prevalence, or proportion of presence to absence points, in our dataset was 0.14.

Our Spearman matrix showed high correlation between distances to development and roads (*r* = 0.78), and slope and ruggedness (*r* = 0.75). We dropped distances to development and ruggedness from our candidate models because we felt that roads and slope more closely reflected the factors influencing the location of farms and were more likely to be available as spatial data elsewhere.

The most important variables for predicting the fine-grained distribution of swine farms in the U.S. were distances to open areas, cropland, and roads, annual mean temperature, and slope. Precipitation, and distances to urban markets, wetlands, forests and barren land were less important but still included in our final model (Table 3).

**Table 3:** Variable importance ranks for individual covariates used for predicting the distribution of swine farms in the conterminous U.S. Each run is an iteration of a 5-fold cross-validation where 80% of the dataset was used for model building and 20% used for model testing. Quadratic forms of these covariates were used when their AIC values were less than the linear forms (S1 Table).

Covariate^a^	Run 1	Run 2	Run 3	Run 4	Run 5	Mean	SE
*d*Open	0.438	0.448	0.449	0.443	0.468	0.449	0.005
*d*Crop	0.223	0.227	0.237	0.227	0.214	0.226	0.004
Temp	0.223	0.234	0.191	0.238	0.230	0.223	0.008
*d*Roads	0.201	0.195	0.215	0.209	0.178	0.200	0.006
Slope^2^	0.141	0.138	0.133	0.133	0.130	0.135	0.002
Precip^2^	0.077	0.059	0.061	0.082	0.056	0.067	0.005
*d*Forest^2^	0.010	0.009	0.013	0.009	0.009	0.010	0.001
*d*Urban^2^	0.010	0.006	0.002	0.007	0.009	0.007	0.001
*d*Wetland^2^	0.010	0.006	0.005	0.004	0.010	0.007	0.001
*d*Barren^2^	0.001	0.003	0.003	0.002	0.001	0.002	< 0.001

^a^ Covariates with prefix *d* are measured as distance to the environmental or anthropogenic feature.

Eight candidate models had AICΔ_*i*_ values ≤ 2 indicating they were equivalent models [25] (Table 4). These eight models were used to estimate our final model-averaged equation:

$$\begin{array}{l} \;\mathrm{logit}\;\left(y\right)=\left(-5.66\times 10^{-3}\times d\mathrm{Open}\right)-(4.12\times 10^{-4}\times d\mathrm{Crop})-\left(8.51\times 10^{-3}\times \mathrm{Temp}\right)-\\ \left(1.48\times 10^{-3}\times d\mathrm{Roads}\right)-\left(2.62\times 10^{-2}\times {\mathrm{Slope}}^{2}\right)+\left(3.29\times 10^{-7}\times {\mathrm{Precip}}^{2}\right)-\\ \left(4.84\times 10^{-9}\times d{\mathrm{Forest}}^{2}\right)-\left(2.95\times 10^{-10}\times d{\mathrm{Urban}}^{2}\right)-\left(1.28\times 10^{-9}\times d{\mathrm{Wetland}}^{2}\right)\\ -\left(2.58\times 10^{-10}\times d{\mathrm{Barren}}^{2}\right)-5.63\times 10^{-2} \end{array}$$

**Table 4:** Model-selection analysis for logistic regression modeling of swine farm distribution in the conterminous U.S. Results are shown for models with *AIC*Δ_*i*_ ≤ 2.0 and all these models were used to develop model-averaged coefficients for our final distribution model.

Model^a^	*LL*	*df*	*AIC*	*AICΔ*_*i*_
*d*Open, *d*Crop, Temp, *d*Roads, Slope^2^, Precip^2^, *d*Forest^2^, *d*Urban^2^, *d*Wetland^2^	-3463.2	10	6946.5	0.0
*d*Open, *d*Crop, Temp, *d*Roads, Slope^2^, Precip^2^, *d*Forest^2^, *d*Urban^2^, *d*Wetland^2^, *d*Barren^2^	-3462.3	11	6946.7	0.2
*d*Open, *d*Crop, Temp, *d*Roads, Slope^2^, Precip^2^, *d*Forest^2^, *d*Wetland^2^, *d*Barren^2^	-3463.5	10	6947.1	0.6
*d*Open, *d*Crop, Temp, *d*Roads, Slope^2^, Precip^2^, *d*Forest^2^, *d*Wetland^2^	-3464.5	9	6947.1	0.6
*d*Open, *d*Crop, Temp, *d*Roads, Slope^2^, Precip^2^, *d*Forest^2^, *d*Urban^2^	-3465.0	9	6947.9	1.4
*d*Open, *d*Crop, Temp, *d*Roads, Slope^2^, Precip^2^, *d*Forest^2^, *d*Urban^2^, *d*Barren^2^	-3464.0	10	6948.0	1.5
*d*Open, *d*Crop, Temp, *d*Roads, Slope^2^, Precip^2^, *d*Forest^2^, *d*Barren^2^	-3465.2	9	6948.4	1.9

^a^ Covariates with prefix *d* are measured as distance to the environmental or anthropogenic feature.

**Fig 5:**
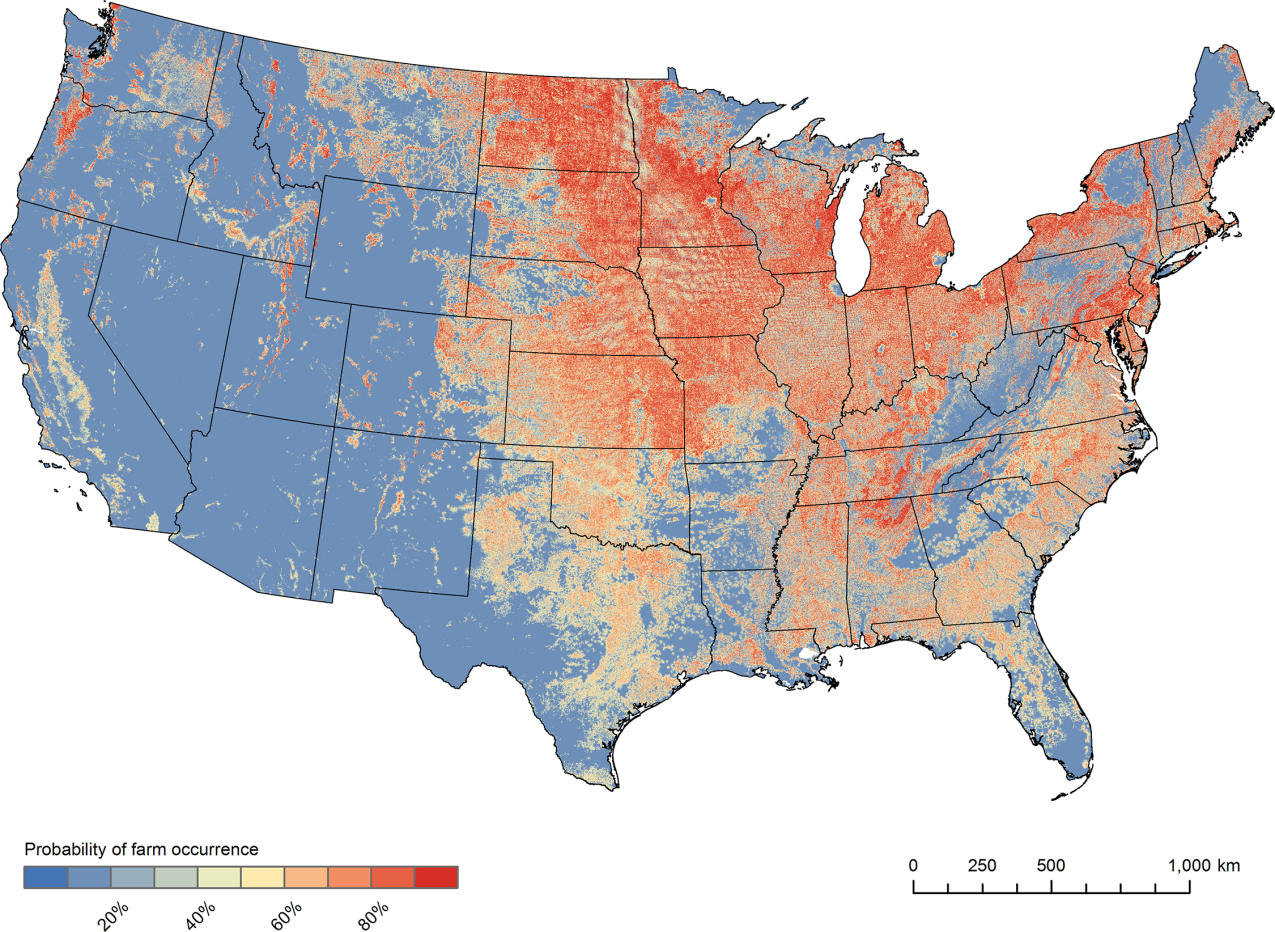
The probability surface used to simulate the locations of individual farms throughout the conterminous United States. The blue to red color scheme represents a gradient of low to high predicted probability values at a 100 m resolution.

We mapped this equation using the Spatial Analyst extension in ArcGIS10.1 [19]. The simulated farm locations were based on the probability surface created from this equation (Fig 5).

Our best candidate model had a mean AUC value of 0.78 across the five folds of our 80:20 testing:training cross-validation. This result was considered good but not excellent based on criteria in Hosmer and Lemeshow [34], indicating that many swine farms did not occur in locations with the highest occurrence probabilities.

### Simulation model for simulating swine populations on individual farms

The simulation model was able to allocate individual swine farms and their populations for the conterminous U.S. in approximately 30 minutes on a computer with a six-core 3.2 GHz processor and 32 GB of memory. The simulation took approximately 60 minutes on a less powerful computer with a dual-core 2.5 GHz processor and 4 MB of memory.

Output data are provided as a comma-separated value file containing the simulated locations and populations for all individual swine farms in the conterminous U.S. (see S1 Dataset for ten example records). Fields in the output data include the FIPS code that uniquely identifies U.S. counties, state name, geographic coordinates of latitude and longitude in decimal degrees, production level (i.e., size of the farm’s livestock population), and commodity (e.g., swine). A web-based graphical-user interface is available so that requests for model runs and data can be initiated by registered end users and FLAPS output can be delivered via email.

The reaggregation analysis for our simulation model verified our algorithms accurately disaggregated the number of individual farms included the aggregate county totals. We found a mean (± *SE*) absolute percentage difference of 0.03 ± 0.01% across all 48 states. The highest mean absolute percent difference was New Jersey (0.6%) and every farm was allocated for seven states (i.e., 0.0% for Arizona, Colorado, Delaware, Mississippi, Nevada, New Mexico, and West Virginia). Summing these individual farms counts to the national-level showed 11 of the 20 runs allocated the exact number of pigs reported in the conterminous U.S. in the 2012 Census of Agriculture (66,015,915), while the other nine runs had a difference of only 1–2 pigs due to rounding errors.

## Discussion

The FLAPS model incorporated several methodological advances that could be useful for developing microsimulation models for other systems. Most notably, we incorporated a probabilistic-distribution model into a microsimulation modeling framework. There has been surprisingly little effort to improve the spatial accuracy of microsimulation models. Models often define geographic locations randomly or with subjective rules. Previous synthetic livestock population models have often simulated the spatial distribution of farms using rules defining where farms cannot occur [10–13]. The ability to use probabilistic spatial predictions to place experimental units in areas with similar characteristics as actual locations should improve the spatial accuracy and realism of microsimulation models.

Our creation of a 10,000 record empirical dataset of farm presences and absences is a key characteristic that separates the FLAPS model from other livestock-simulation models. Presence-absence data are widely used in biogeography and ecology to predict the distribution of organisms [32,35,36]. Similar to our study, Neumann et al. [10] used data from an agricultural census in Europe to predict the distribution of livestock at a 1 km^2^ resolution for that region. However, their study did not sample the location of individual farms and instead relied upon coarser resolution data. The density of poultry production in China was similarly modeled with coarse-grained aggregate data [12]. While Emelyanova et al. [11] did sample data at individual cattle farms in Australia, they built their models with a smaller dataset of farm locations (*n* = 217) than our study and did not specifically sample areas lacking farms (i.e., absence points). The inclusion of absence information improves the performance of distribution models [32]. Our use of a large empirical dataset allowed us to better describe the geographical patterns of farm location and allowed FLAPS to have a finer granularity than most previous livestock population simulators (100 m vs.1 km resolution) [10–12]. The good but not excellent results of our geographic validation were not surprising given that the fine-grained geographical distribution of swine farms is a highly variable system influenced by many spatial, temporal, economic and historical factors.

The covariates we used in our distribution model reflected our interest in predicting farm locations at a 100 m resolution. For example, most of our covariates were measured as the distance to landscape features or land-cover classes, a tactic that has been similarly applied to study the movement of animals at fine spatial scales [37]. Distances were especially useful in this study since, like studies of animal movement, they provided a better measure of how farms were distributed relative to linear features like roads and the edges of land-cover types, while still accounting for whether the data were distributed within or outside of area-based features like patches of land cover [37]. Our finding of clear patterns of livestock farms being located closer to features like roads suggests that earlier livestock population simulators that utilized distance-based rules may do a reasonable job of capturing the variability inherent in the geography of farm locations, but empirical data in probabilistic models should improve results [10]. The strong associations we saw between farm locations and land-cover classes like cropland or forests corroborate the results of a recent study that found land cover was important for understanding the fine-grained geography of farm locations in the UK [38]. Overall, we found clear differences in the characteristics of locations where farms occurred and where they did not occur. These results indicate that population simulation models for livestock should incorporate realistic distribution information to accurately depict the fine-grained geography of farm locations. A failure to account for this geography could negatively affect applications of models like FLAPS. For example, random placement of farms negatively impacted the outcome of an epidemiological model of foot-and-mouth disease [38].

In addition to incorporating a distribution model to improve geographic accuracy of model output, we also addressed two demographic challenges concerning how to impute the missing aggregate Census of Agriculture data and then simulate a synthetic dataset of swine farms and their locations and populations. While these are common challenges when developing microsimulation models, the characteristics of the U.S. Census of Agriculture dataset required us to modify existing methods. We discuss these modifications in more detail for others facing similar challenges.

Our first challenge was how to estimate any missing county-level pig population totals or subtotals in the seven-binned frequency distribution that depicted each county’s pig population. Certain features of the Census of Agriculture provided key information that allowed us to impute realistic missing data. First, the hierarchical structure of the Census of Agriculture provided marginal totals for our IPF algorithms. Specifically, the state totals provided marginal column totals for our county-level missing-data model, and the national totals provided marginal column totals for our state-level missing-data model. However, we needed to impute missing data in our marginal totals. The key feature of the Census of Agriculture that facilitated imputation was access to the frequency distributions of cross-classified farm by population data. Except for the largest bin with an unbounded maximum, the bins of the frequency distribution provided a range of possible values for any missing data within the frequency distribution or, after summing all seven bins, marginal totals. The range of possible values could be further refined because the frequency distributions describing the number of farms in each bin were always published and had the same bin sizes. Using the number of farms as a multiplier, we obtained a known and unambiguous range of possible values for the aggregate county-level totals and subtotals. These ranges were subsequently refined with our IPF algorithms. We found that the information provided by the hierarchical structure of the Census data, access to partial frequency distributions for swine populations, and complete farm frequency distributions for the number of swine farms provided a better imputation methodology than even a spatially-explicit probabilistic method like geographically-weighted regression, where estimates were frequently outside the known range of possible values.

We only needed to impute missing data for the swine population. The ability to use IPF algorithms without microsample data becomes more complex when multiple variables are needed in the simulated output. For example, future development of the FLAPS model will likely include refining our baseline species-level output for classification into specific production types, which typically are age or breeding classes in the swine industry. Some recent methods that have simulated individual-level data over several cross-classified variables without a data microsample may help guide this refinement [33,39].

The final step was to use similar iterative-fitting algorithms to disaggregate our complete aggregate dataset and create a synthetic population of pig populations on individual farms. Our simulated aggregate Census of Agriculture dataset of paired frequency distributions output from our missing-data model provided input for this step. We simply applied a uniform distribution and simulated realistic values for individual farms by distributing the total pig population across the known number of swine farms.

It is important that end users of FLAPS output are aware of the demographic and geographic uncertainty in model output. While a validation of our final farm-level output with external data was not possible since such data were unavailable at a national scale, our approach restricted the demographic variation between actual and simulated farm-level data to the corresponding bin sizes in the aggregate Census data (1–24, 25–49, 50–99, 100–199, 200–499, 500–999, and ≥ 1000 pigs). Consequently, the demographic uncertainty of FLAPS output is dependent on, and increases with, the population size of the farm, but errors are also minimized by the constraint that they occur within the range of their bin of the aggregate frequency distributions. Note that FLAPS allocates data directly from the Census of Agriculture and does not address any demographic uncertainty in the Census. The finest resolution aggregate data available in the Census was for individual U.S. counties, so this county-level resolution defines the geographic uncertainty of FLAPS output. Caution should therefore be used if using FLAPS output for analyses for a single or small number of U.S. counties in states with small swine industries since these regions typically have more redacted data. We recommend using output from multiple FLAPS runs to better account for any demographic and geographic uncertainty.

The output from FLAPS has many uses but should be particularly valuable by providing an essential infrastructural element for managing risks to animal and human health. Although our output should ideally be supplemented with additional information about transmission pathways, the output of FLAPS can facilitate epidemiological studies of swine diseases, some of which have direct impacts on human health. Pigs are known vectors of influenza viruses and can be infected from either birds (wild and domestic) or humans [40–42]. They are also known carriers of the highly pathogenic H5N1 avian influenza virus [43]. Pigs can serve as mixing vessels where reassortment of avian, human, and swine influenza viruses can create novel genetic variants [40]. Much of the testing for influenza in domestic pigs is performed by the U.S. swine industry and this information is not widely available for management and research [44]. The swine industry, like other livestock industries in the U.S., has become increasingly geographically concentrated [9]. Some of the recent growth in the hog industry has occurred in non-traditional regions [9], which may lack the necessary veterinary infrastructure to prevent and manage the spread of diseases. One of the key risk factors influencing disease spread is the spatial proximity of potential host populations [38], making knowledge of the distances between farms critical information for controlling the spread of disease. The fine-grained spatial structure of farm locations and populations provided by FLAPS can also facilitate the use of farm animals as model systems for understanding human epidemiology [45]. Given that data are unavailable in the U.S., these potential uses underscore the importance of and need for the simulated location and population data that FLAPS provides.

Output from FLAPS can also inform the management of other livestock-related issues. Data depicting the simulated locations and populations of livestock farms are necessary to develop effective emergency-response plans for disease outbreaks, bioterrorism, or disasters like floods and fires. Larger operations can be point sources of pollution, and even simulated location and population information may help reduce the potential contamination of water sources. In a general sense, simulated livestock distribution data should allow us to more effectively study and resolve many problems associated with the overlap of humans, livestock, or wildlife.

We plan several future improvements to the FLAPS model. Modules for other livestock species such as poultry, dairy, beef, sheep and goats are being developed. Also, epidemiological studies would benefit from having additional demographic information like age- or sector-specific production types. Lastly, another potential improvement to the FLAPS model is that it could be adapted to predict the distributions of other agricultural commodities like produce farms.

In conclusion, the Census of Agriculture is the most comprehensive source of agricultural production data for the U.S. and provided us with the best available baseline data to develop a national-scale microsimulation model of livestock distribution. The methodologies we developed to downscale the Census data to individual livestock farms provide a useful template for the development of similar population-simulation models in other regions where comprehensive, aggregated agricultural-production data are available. The ability to develop sustainable livestock-production systems that meet increasing human demand for animal protein while also ensuring animal and human health likely requires access to spatially-explicit, fine-resolution data on the distribution of livestock. The continuing globalization of food-production systems means the importance of understanding the geographical distribution of livestock production expands beyond local or even national boundaries.

## Supporting Information

S1 Dataset A ten record example dataset showing output from the FLAPS model. (CSV)

S1 Presentation Using iterative-proportional fitting to estimate missing data in the Census of Agriculture. (PDF)

S1 Table Model-selection results comparing linear and quadratic forms of covariates used in the FLAPS model. (PDF)

S2 Table Linear distance differences among species-level farm estimates. (PDF)

S1 Text Criteria used to sample for the presence or absence of livestock farms. (PDF)

S2 Text Methods for evaluating differences between species estimated to be present on livestock farms. (PDF)
