# Supplementary material for: Simulating the Distribution of Individual Livestock Farms and Their Populations in the United States: An Example Using Domestic Swine (Sus scrofa domesticus) Farms
Source: PLoS One. 2015 Nov 16;10(11):e0140338. doi: 10.1371/journal.pone.0140338 (PMC4646625; doi:10.1371/journal.pone.0140338)
Supplement: S1 Presentation — (PDF) [file pone.0140338.s002.pdf]

Iterative Proportional Fitting

FIPS	Farm Total	Pop Total	Farms 1000+	Pop 1000+	Farms 500-999	Pop 500-999	Farms 200-499	Pop 200-499	Farms 100-199	Pop 100-199	**Row Totals**	**Row Error**
STATE	62	34,975	10	22,873	4	2,875	8	2,720	38	6,507		
CNTY1	13	9,636	7	8,570	0	0	1	???	5	736		
CNTY2	41	23,489	3	14,303	3	???	5	???	30	5,456		
CNTY3	6	1,850	0	0	1	600	2	???	3	???		
**Col Totals**												
**Col Error**												

Example of CoA data, unpublished values highlighted.

Iterative Proportional Fitting

FIPS	Farm Total	Pop Total	Farms 1000+	Pop 1000+	Farms 500-999	Pop 500-999	Farms 200-499	Pop 200-499	Farms 100-199	Pop 100-199	**Row Totals**	**Row Error**
STATE	62	34,975	10	22,873	4	2,875	8	2,720	38	6,507		
CNTY1	13	9,636	7	8,570	0	0	1	???	5	736		
CNTY2	41	23,489	3	14,303	3	???	5	???	30	5,456		
CNTY3	6	1,850	0	0	1	600	2	???	3	???		
**Col Totals**												
**Col Error**												

Information aggregated at the state level.

Iterative Proportional Fitting

FIPS	Farm Total	Pop Total	Farms 1000+	Pop 1000+	Farms 500-999	Pop 500-999	Farms 200-499	Pop 200-499	Farms 100-199	Pop 100-199	**Row Totals**	**Row Error**
STATE	62	34,975	10	22,873	4	2,875	8	2,720	38	6,507		
CNTY1	13	9,636	7	8,570	0	0	1	???	5	736		
CNTY2	41	23,489	3	14,303	3	???	5	???	30	5,456		
CNTY3	6	1,850	0	0	1	600	2	???	3	???		
**Col Totals**												
**Col Error**												

Information aggregated at the county level.

Iterative Proportional Fitting

FIPS	Farm Total	Pop Total	Farms 1000+	Pop 1000+	Farms 500-999	Pop 500-999	Farms 200-499	Pop 200-499	Farms 100-199	Pop 100-199	**Row Totals**	**Row Error**
STATE	62	34,975	10	22,873	4	2,875	8	2,720	38	6,507		
CNTY1	13	9,636	7	8,570	0	0	1	???	5	736		
CNTY2	41	23,489	3	14,303	3	???	5	???	30	5,456		
CNTY3	6	1,850	0	0	1	600	2	???	3	???		
**Col Totals**												
**Col Error**												

Overall totals for state and county level data.

Iterative Proportional Fitting

FIPS	Farm Total	Pop Total	Farms 1000+	Pop 1000+	Farms 500-999	Pop 500-999	Farms 200-499	Pop 200-499	Farms 100-199	Pop 100-199	**Row Totals**	**Row Error**
STATE	62	34,975	10	22,873	4	2,875	8	2,720	38	6,507		
CNTY1	13	9,636	7	8,570	0	0	1	???	5	736		
CNTY2	41	23,489	3	14,303	3	???	5	???	30	5,456		
CNTY3	6	1,850	0	0	1	600	2	???	3	???		
**Col Totals**												
**Col Error**												

Aggregated population groups.

Iterative Proportional Fitting

FIPS	Farm Total	Pop Total	Farms 1000+	Pop 1000+	Farms 500-999	Pop 500-999	Farms 200-499	Pop 200-499	Farms 100-199	Pop 100-199	**Row Totals**	**Row Error**
STATE	62	34,975	10	22,873	4	2,875	8	2,720	38	6,507		
CNTY1	13	9,636	7	8,570	0	0	1	???	5	736		
CNTY2	41	23,489	3	14,303	3	???	5	???	30	5,456		
CNTY3	6	1,850	0	0	1	600	2	???	3	???		
**Col Totals**												
**Col Error**												

Marginal totals for the rows and columns.

Iterative Proportional Fitting

FIPS	Farm Total	Pop Total	Farms 1000+	Pop 1000+	Farms 500-999	Pop 500-999	Farms 200-499	Pop 200-499	Farms 100-199	Pop 100-199	**Row Totals**	**Row Error**
STATE	62	34,975	10	22,873	4	2,875	8	2,720	38	6,507		
CNTY1	13	9,636	7	8,570	0	0	1	393	5	736		
CNTY2	41	23,489	3	14,303	3	2,100	5	1,600	30	5,456		
CNTY3	6	1,850	0	0	1	600	2	699	3	450		
**Col Totals**												
**Col Error**												

Use population group information to generate seed values.

Iterative Proportional Fitting

FIPS	Farm Total	Pop Total	Farms 1000+	Pop 1000+	Farms 500-999	Pop 500-999	Farms 200-499	Pop 200-499	Farms 100-199	Pop 100-199	**Row Totals**	**Row Error**
STATE	62	34,975	10	22,873	4	2,875	8	2,720	38	6,507		
CNTY1	13	9,636	7	8,570	0	0	1	393	5	736		
CNTY2	41	23,489	3	14,303	3	2,100	5	1,600	30	5,456		
CNTY3	6	1,850	0	0	1	600	2	699	3	450		
**Col Totals**												
**Col Error**												

Add across each row and down each column...

Iterative Proportional Fitting

FIPS	Farm Total	Pop Total	Farms 1000+	Pop 1000+	Farms 500-999	Pop 500-999	Farms 200-499	Pop 200-499	Farms 100-199	Pop 100-199	**Row Totals**	**Row Error**
STATE	62	34,975	10	22,873	4	2,875	8	2,720	38	6,507		
CNTY1	13	9,636	7	8,570	0	0	1	393	5	736	9,699	
CNTY2	41	23,489	3	14,303	3	2,100	5	1,600	30	5,456	23,459	
CNTY3	6	1,850	0	0	1	600	2	699	3	450	1,749	
**Col Totals**				22,873		2,700		2,692		6,642		
**Col Error**												

…to get the current row/column totals…

Iterative Proportional Fitting

FIPS	Farm Total	Pop Total	Farms 1000+	Pop 1000+	Farms 500-999	Pop 500-999	Farms 200-499	Pop 200-499	Farms 100-199	Pop 100-199	**Row Totals**	**Row Error**
STATE	62	34,975	10	22,873	4	2,875	8	2,720	38	6,507		
CNTY1	13	9,636	7	8,570	0	0	1	393	5	736	9,699	63
CNTY2	41	23,489	3	14,303	3	2,100	5	1,600	30	5,456	23,459	30
CNTY3	6	1,850	0	0	1	600	2	699	3	450	1,749	101
**Col Totals**				22,873		2,700		2,692		6,642		
**Col Error**				0		175		28		135		

…and the marginal errors for each row and column.

Iterative Proportional Fitting

FIPS	Farm Total	Pop Total	Farms 1000+	Pop 1000+	Farms 500-999	Pop 500-999	Farms 200-499	Pop 200-499	Farms 100-199	Pop 100-199	**Row Totals**	**Row Error**
STATE	62	34,975	10	22,873	4	2,875	8	2,720	38	6,507		
CNTY1	13	9,636	7	8,570	0	0	1	394	5	736	9,700	64
CNTY2	41	23,489	3	14,303	3	2,239	5	1,593	30	5,456	23,591	102
CNTY3	6	1,850	0	0	1	600	2	737	3	456	1,793	57
**Col Totals**				22,873		2,839		2,724		6,648		
**Col Error**				0		36		4		141		

After one iteration of the IPF method.

Iterative Proportional Fitting

FIPS	Farm Total	Pop Total	Farms 1000+	Pop 1000+	Farms 500-999	Pop 500-999	Farms 200-499	Pop 200-499	Farms 100-199	Pop 100-199	**Row Totals**	**Row Error**
STATE	62	34,975	10	22,873	4	2,875	8	2,720	38	6,507		
CNTY1	13	9,636	7	8,570	0	0	1	330	5	736	9,636	0
CNTY2	41	23,489	3	14,303	3	2,275	5	1,455	30	5,456	23,489	0
CNTY3	6	1,850	0	0	1	600	2	935	3	315	1,850	0
**Col Totals**				22,873		2,875		2,720		6,507		
**Col Error**				0		0		0		0		

After multiple iterations of the IPF method.
