# Supplementary material for: Simulating the Distribution of Individual Livestock Farms and Their Populations in the United States: An Example Using Domestic Swine (Sus scrofa domesticus) Farms
Source: PLoS One. 2015 Nov 16;10(11):e0140338. doi: 10.1371/journal.pone.0140338 (PMC4646625; doi:10.1371/journal.pone.0140338)
Supplement: S1 Table — (PDF) [file pone.0140338.s003.pdf]

S1 Table. A comparison of relative model quality between simple linear and squared quadratic version of our geographic-model covariates. The best version of the covariate is highlighted in bold.

Covariate	AIC_{Linear}	$AIC_{Quadratic}$	$AIC\Delta_i$
Barren	8109.0	**8106.7**	2.3
Crop	**7718.1**	7852.9	134.7
Forest	8085.7	**8074.1**	11.6
Open	**7647.5**	7726.9	79.4
Pasture	**8073.3**	8101.7	28.4
Urban	8053.9	**8023.2**	30.7
Wetland	8080.4	**8074.1**	6.3
Roads	**7702.9**	7764.7	61.8
Temp	**7945.7**	8000.2	54.5
Precip	8107.6	**8093.1**	14.5
Slope	7945.7	**7900.0**	45.8
