# Supplementary material for: Simulating the Distribution of Individual Livestock Farms and Their Populations in the United States: An Example Using Domestic Swine (Sus scrofa domesticus) Farms
Source: PLoS One. 2015 Nov 16;10(11):e0140338. doi: 10.1371/journal.pone.0140338 (PMC4646625; doi:10.1371/journal.pone.0140338)
Supplement: S2 Table — (PDF) [file pone.0140338.s004.pdf]

Table S2. The mean (± SE) differences (values are distances in meters) and results of an ANOVA (*P*-value) comparing the differences among our three best distance-based covariates for the different farm types defined in our sampling design. Although the ANOVA indicates these differences are significant, they are generally below the 100 m resolution of the FLAPS model. We therefore did not classify farms to the species suspected to be present given the greater uncertainty of these species-level classifications.

**Variable**	**Pigs**	**Poultry**	**Other**	**Corrals**	***P*-value**
*d*Open	111 ± 9	92 ± 6	50 ± 5	38 ± 2	< 0.001
*d*Crop	129 ± 37	537 ± 78	191 ± 33	317 ± 32	< 0.001
*d*Roads	246 ± 17	173 ± 10	172 ± 11	158 ± 7	< 0.001
