# Supplementary material for: Simulating the Distribution of Individual Livestock Farms and Their Populations in the United States: An Example Using Domestic Swine (Sus scrofa domesticus) Farms
Source: PLoS One. 2015 Nov 16;10(11):e0140338. doi: 10.1371/journal.pone.0140338 (PMC4646625; doi:10.1371/journal.pone.0140338)
Supplement: S1 Text — (PDF) [file pone.0140338.s005.pdf]

S1 Text.

Sampling to determine the presence or absence of livestock farms was based on a hierarchical decision tree (Figure 1, Text S1). The technician first decides if a farm is present in a sampling cell, then determines if livestock infrastructure is present, and finally estimates the livestock species likely to be present on the farm. We defined livestock infrastructure as any feature like barns, corrals, or feed storage bins that need to be present at locations where poultry or livestock (pigs, beef cattle, dairy cattle, sheep, goats, or horses) are being raised. We found it was simple to detect such livestock infrastructure with aerial photography, but there is greater uncertainty in the species-level estimates.

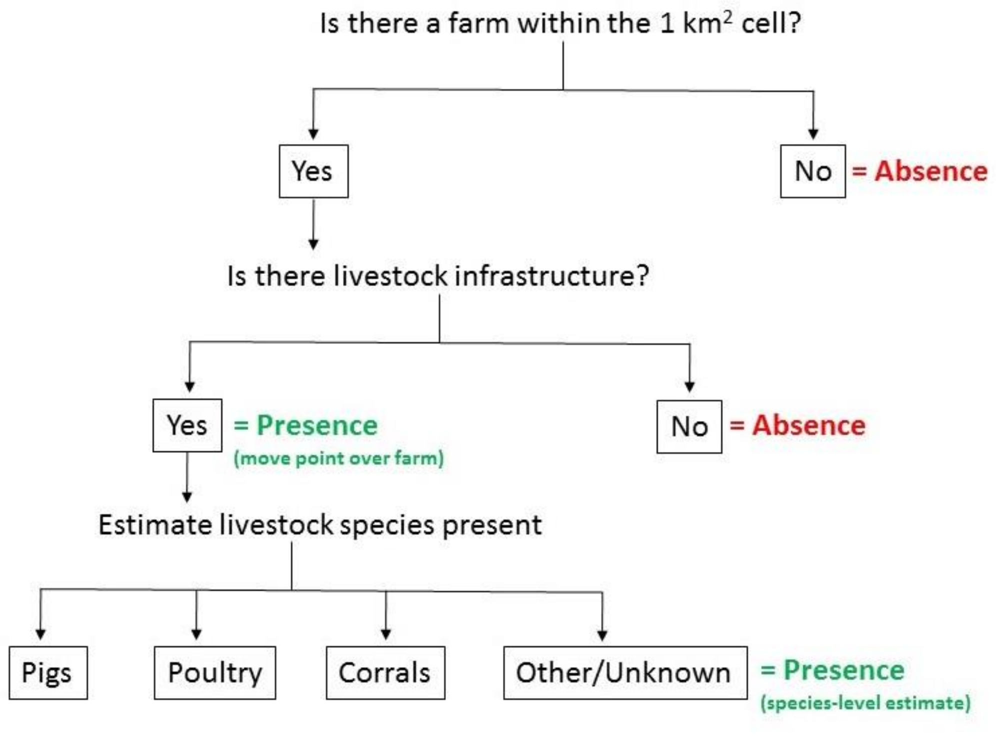

Figure 1, Text S1. Decision tree to identify livestock farms and estimate species being raised at the site.

The process we used to estimate the livestock species present was based on criteria developed through interviews with livestock industry experts. The experts we interviewed were based out of the United States Department of Agriculture, Animal and Plant Health Inspection Service, Veterinary Services (USDA/APHIS/VS), Center for Epidemiology and Animal Health (CEAH), Fort Collins, Colorado. Our interviewees included Eric Bush (pigs), Dave Dargatz (beef cattle), Lindsey Garber (poultry), Jason

Lombard (dairy cattle), Reginald Johnson (sheep and goats), and Katherine Marshall (sheep and goats).

The criteria obtained from our interviews are summarized below. Because our sampling design was focused on detecting swine farms in the major swine producing regions, in this study we did not attempt to define larger cattle farms, or farms raising horses, sheep, or goats. However, we are currently conducting similar sampling designs for beef cattle, dairy cattle, and poultry. We anticipate this broader sample will allow us to better estimate the species being raised at livestock farms.

Poultry

Presence of row barns - long and thin barns, often several parallel or close together (poultry barns usually narrower and sometimes longer than swine barns)

Ground around barns is kept free to vegetation, equipment, and other clutter.

Swine

Presence of row barns - long and thin barns, often several parallel or close together (swine barns usually wider and longer than poultry barns)

Presence of manure lagoon

Almost always have “unnatural” shape [e.g., rectangle] and manure color

Lagoon perhaps strongest evidence of a swine (rather than poultry) operation so if present classify as swine even if other characteristics (e.g., distance from roads, etc.) are inconclusive

Presence of silos (structures for holding feed near barns) likely swine (especially if large) rather than poultry

Connections between row barns (enclosed structure to constrain movement) associated with swine

Swine barns often slightly farther from road/human residence than poultry (reduce noise/odor)

Presence of windbreaks (trees planted in rows to hide barns), reduce noise or odor)

Corral

Corral is fenced-in outdoor area in which livestock is kept. Smaller corrals often have bare soil, whereas larger corrals are essentially pastures with grass.

Animal trails are common around feeding sites.

Corral is key attribute to identifying smaller livestock (pigs, cattle, etc.) operations

Other/Unknown

This category includes farms raising dairy cattle, beef cattle (larger facilities), horse, other ruminants like sheep or goats, or other unknown species.

Because of the corral category, farms in this category tend to have larger livestock populations.
