# Supplementary material for: Simulating the Distribution of Individual Livestock Farms and Their Populations in the United States: An Example Using Domestic Swine (Sus scrofa domesticus) Farms
Source: PLoS One. 2015 Nov 16;10(11):e0140338. doi: 10.1371/journal.pone.0140338 (PMC4646625; doi:10.1371/journal.pone.0140338)
Supplement: S2 Text — (PDF) [file pone.0140338.s006.pdf]

We conducted an analysis of variance (ANOVA) to evaluate if covariate values differed among the various livestock species presumed present at our presence points. If significant difference were found, we considered effect sizes to determine if the differences were relevant given the 100 m resolution of FLAPS output. We were concerned that statistically significant differences might arise as a consequence of our large sample, but that these differences would be near the resolution of the FLAPS model. We assumed if the effect sizes among the livestock-species categories were near the 100 m resolution of our model that all presence records could be used to predict the distribution of swine farms. Similar to the use of distribution models in ecology [13], we used the presence-absence data as the binary response variable in a logistic-regression model.

We found statistically significant differences among the top covariates for the different types of livestock farms in our sample (S2 Table). However, these differences were mostly artifacts of our large sample size. Overall, the effect of these differences would not impact our geographic simulation of farm locations because the differences among farm types were usually below the 100 m resolution of our model (S2 Table). Given the additional uncertainty associated with making species-level classifications of farm type based on infrastructure, the inability to determine species present at the smaller farms with corrals, and the potential for introducing bias if only farms with typical characteristics of large swine operations were used for predictions, we used all livestock farms in our sample to predict the geographic distribution of swine farms.
